# Personalized infection prevention and control: a concept whose time has arrived

**DOI:** 10.1017/ash.2023.429

**Published:** 2023-09-08

**Authors:** Alessia Savoldi, Nico T. Mutters, Evelina Tacconelli

**Affiliations:** 1 Division of Infectious Diseases, Department of Diagnostics and Public Health, University of Verona, Verona, Italy; 2 Institute for Hygiene and Public Health, University Hospital Bonn, Bonn, Germany; 3 ESCMID European Committee on Infection Prevention and Control (EUCIC), Basel, Switzerland

**Keywords:** hospital transmission, infection prevention, personalized medicine

## Abstract

Personalized medicine has been progressively implemented in several diagnostic and therapeutic patients’ algorithms, based on the common assumption that tailoring interventions, practices, and/or therapies to individual patients’ clinical, biological, epidemiological, and genetic characteristics would optimize their effectiveness and reduce adverse effects. The potential benefit of the precision medicine approach has been recently considered for possible implementation in the field of infection prevention and control. The commentary explores available evidence and assesses possible future scenarios where, through advanced modeling approaches, we would be able to provide personalized prediction algorithms identifying at-risk patients who deserve the implementation of tailored preventive measures.

## Introduction

Personalized medicine, also referred to as precision medicine, has been progressively implemented in several diagnostic and therapeutic patients’ algorithms for noncommunicable diseases, based on the common assumption that tailoring interventions, practices, and/or therapies to individual patients’ clinical, biological, epidemiological, and genetic characteristics would optimize their effectiveness.^1,2^ Oncology represents the medical area where this concept has been incorporated on a wide scale into treatment and prevention programs in the context of primarily three determinants: lifestyle, comorbidities, and genomic and epigenomic profiling.^3^ In the area of infectious diseases, a recent example of personalized medicine application emerged during the Coronavirus Disease 19 pandemic, where clinical data coupled with genomics and molecular technologies were utilized for identifying etiologic agents, developing diagnostics and treatments, and creating vaccine candidates.^1^

**Figure 1.**
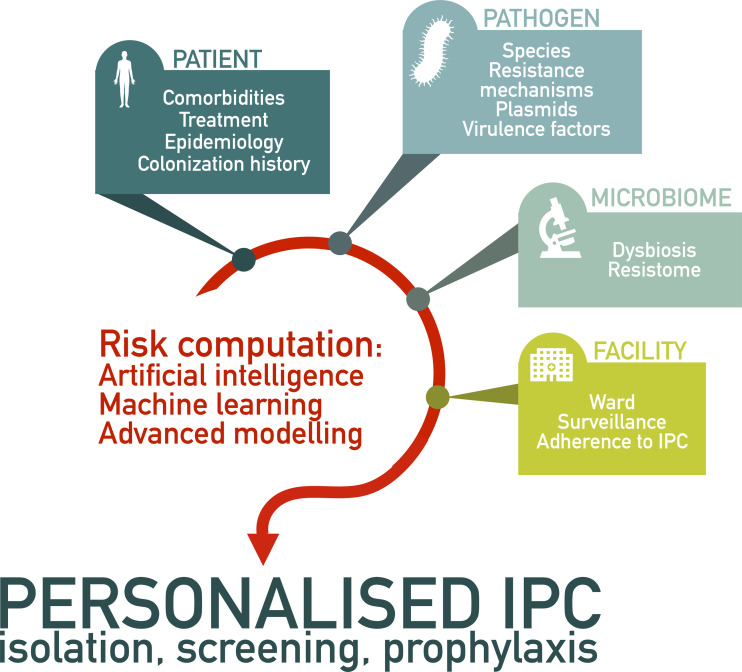
Integration of multiple data sources to personalize the implementation of infection prevention and control measures.

The potential benefit of the precision approach has been recently considered for possible implementation in the field of infection prevention and control (IPC).^4,5^ The ideal goal of precision IPC would be to implement a measure, or a bundle of measures, specifically in selected individuals considered at-risk on the basis of patient-related (epidemiology, comorbidities, omics profile) and pathogen-related (molecular resistance mechanism and/or virulence factors) determinants (Figure 1). The ability to precisely quantify the risk of horizontal transmission at individual-level in colonized and/or infected patients would lead not only to an optimized effectiveness of the implemented measure but possibly also to a decrease of adverse effects (such as reduced contact with healthcare workers, organizational constraints due to single room isolation) of hospital personnel’s workload, and of hospital costs. In parallel, the reduced workload could lead to an improvement in adherence to interventions and, therefore, a potential further reduction in horizontal transmission among patients and/or healthcare workers.

### Current applications of personalized IPC

The process toward personalization of IPC is, nevertheless, extremely challenging in terms of feasibility, sustainability, transferability, and quality of the available evidence. The issue of how to properly apply precision medicine to IPC has recently arisen from new evidence, which questions and reconsiders the universal use of contact precautions (CP) for inpatients with multi-drug resistant microorganisms (MDROs) outside the outbreak context.^6^ The most advanced discussion focuses on patients colonized or infected by extended β lactamase-producing Enterobacterales (ESBL-E). The currently available IPC guidelines strongly recommend the implementation of CP in endemic settings for inpatients colonized or infected with ESBL-E,^7,8^ except for *Escherichia coli* because of insufficient data allowing to draw evidence-based conclusions in high-risk patients.^7^ More recently, a 2023 scoping review of nine studies (one of them with randomized design^9^) confirmed no benefit in implementing contact over standard precautions in hospital transmission of ESBL-E.^10^ These results should, however, be cautiously interpreted in terms of generalizability due to high heterogeneity of colonization pressure among wards, healthcare workload organization, hospital structure, IPC compliance, and population type. For example, no studies targeted high-risk settings, while almost all studies were conducted in endemic areas where the community reservoir, especially for *E. coli*, was probably the primary driver for transmission.^11^

The availability of information related to the colonization status and, therefore, the decision as to whether to apply IPC strategies or not is strictly connected to the screening policies (e.g., timing, and universal vs targeted populations). The topic is not straightforward considering that the screening has not only an IPC value but also a clinical relevance providing essential information to drive, for example, the empiric antibiotic therapy in high-risk populations^12^ or surgical peri-operative antibiotic prophylaxis (PAP). The majority of post-operative surgical site infections (SSIs) are indeed caused by endogenous flora and several observational studies confirmed a link between infection and patients’ own microbiota.^13^ The 2023 ESCMID/EUCIC guidelines indicate to implement rectal screening for identifying ESBL-E carriers before colorectal and liver transplant surgery to adapt PAP accordingly.^14^ The recommendation is conditional and the evidence supporting its benefit in reducing post-operative SSIs is represented by few studies with several inherent limitations such as non-randomized design, sample size, and selection and ascertainment biases.^15–19^ However, the translation of this recommendation on personalized PAP into clinical practice raises several difficult IPC questions related to pre-surgical workload organization, laboratory capability, and related costs. In this case, PAP personalization at patient-level should possibly consider the intestinal microbiome composition, which, in the case of patients undergoing colorectal surgery, has been shown to be extremely dynamic over time.^20^ The gut microbiome can mediate colonization resistance against several enteric pathogens through several mechanisms such as nutrient competition, production of antimicrobial compounds, support of gut barrier integrity, bacteriophage deployment, and interaction with the immune system.^21^ According to recent multi-omics microbiome analyses, asymptomatic gut microbiome-mediated colonization resistance is less relevant for ESBL *E.coli* compared to other MDROs and, therefore, microbiome-based interventions might not be the way forward to prevent intestinal colonization of ESBL *E.coli*.^22^

### The future state

A futuristic personalized implementation of IPC measures might be based on longitudinal sampling of a patient’s evolving microbiome to capture pathogens likely to be transmitted and enables for a dynamic modulation of IPC across the whole hospital stay, with possible positive consequences also from an antimicrobial stewardship perspective. The evaluation of the microbiome might be of particular importance for decision-making in case of hematological patients and those undergoing colorectal or transplant surgery, in which the colonization with ESBL-E usually precedes the infection.

As stated above, the precision approach at a microbiological level should also consider if the presence of specific resistance mechanisms such as *AmpC* β lactamase vs OXA-48 carbapenemase would have a role in IPC measure selection, and the evidence here is extremely poor. From a microbiological point of view, resistance mechanisms such as OXA-48-like carbapenemases are often found on the same plasmid that can also be transferred to other bacterial species, although there are multiple plasmids able to carry these mechanisms of resistance. For example, OXA-181 and OXA-232 are associated with ISEcp1, Tn2013 on ColE2, and IncX3 types of plasmids; therefore, the clonal dissemination plays a minor role in the spread of OXA-48-like carbapenemases.^23,24^ Furthermore, the virulence properties implicated in the effectiveness of transmission are not necessarily associated with the plasmid carrying the resistance gene.^24^ On the basis of these considerations, several factors may impact the risk of transmission and infection and the identification of the resistance mechanism alone cannot be currently considered as sufficient to drive IPC selection.

With increasing genetic data about resistance and virulence genes that are detected in certain endemically found plasmids or associated with certain high-risk clones (e.g., K*lebsiella pneumoniae* sequence type 147 [ST147], ST307, ST15, and ST14 and *E. coli* ST38 and ST410), it can be hypothesized that personalized IPC based on molecular identification of high-risk plasmids or clones will be possible in the future. To achieve this goal, sequencing data should be made publicly and freely available within datasets to ensure continuing identification of newly appearing clones and plasmids.^25^

If we could improve the identification of patients’ and pathogens’ genomic characteristics associated with increased transmission risk in the presence of specific comorbidities and/or treatments, such as chemotherapy in hematological diseases, we would probably start building a completely different approach to IPC. Although patient-level risk factors, in particular for acquiring an MDRO colonization, have been clearly identified in literature (e.g., previous antibiotic treatment, indwelling devices, international travel), if considered individually, these show suboptimal accuracy in identifying patients at higher risk for transmitting or acquiring bacteria colonization during hospitalization. In a Dutch tertiary hospital, a universal risk assessment based on the use of a six-item questionnaire upon hospital admission underestimated the number of MDROs carriers deserving pre-emptive isolation, leading to a total of 1436 days of unjustified isolation in case of false positive assessment.^26^ An increase in prediction accuracy might be pursueded by applying new complex analyses such as machine learning, which may allow the quantification of the impact of each contributing factor within the whole patient risk profile. A multidimensional model, including patient-level data (number of antibiotics, combinations, and sequential usage), was translated into a simple ranking of antibiotics associated with ESBL-E colonization in 12 mixed wards across three European hospitals. The study found that monotherapy with a cephalosporin ranked first in promoting carriage, but the ranking strongly changed in accordance with the sequential usage of antibiotics in the previous 30 days.^27^

Data driven predictive models or algorithms for risk assessment developed using machine learning or similar analytic approaches, which integrate both evidence-based, patient-, pathogen-, and facility-level data might increase the prediction accuracy and help clinicians’ decision-making in identifying which patient is at-risk and deserves, therefore, tailored IPC (isolation, screening) or antibiotic treatment or prophylaxis. At same time, *omic*-based techniques may be employed, in a context of translational medicine, in defining the individual microbiota composition and targeting personalized IPC measures accordingly. To maximize its performance, the individualized approach would require to combine outputs from clinical studies, modeling statistics, cost analysis, and basic research.

We urgently need the implementation of patient-centered IPC measures based on a precision approach; we are definitely not there yet but supporting translation research in the IPC field and integration with machine learning data-driven models could bring us closer to this objective than expected.
